# Urinary DNA methylation biomarkers for bladder cancer diagnosis: a single center case-control study

**DOI:** 10.3389/fonc.2026.1812094

**Published:** 2026-05-13

**Authors:** Chao Li, Rongjin Fang, Jiqian Wang, Yongchao Wang, Songtao Zhao, Qinglong Wu, Chen Ying, Minghuang Rao

**Affiliations:** Department of Urology, Xiamen Haicang Hospital, Xiamen, Fujian, China

**Keywords:** bladder cancer, Hand2, TRPS1, urinary DNA methylation, ZNF154

## Abstract

**Background:**

Noninvasive detection of bladder cancer remains challenging because many urine tests trade sensitivity for specificity. We evaluated whether a biologically grounded, complementary three−gene methylation panel measured in urine could improve diagnostic performance.

**Methods:**

We conducted a single-center 1:1 matched case-control study using clinically relevant urine samples. Urine DNA was subjected to bisulfite conversion and quantitative methylation analysis targeting three gene loci (*TRPS1*, *HAND2*, *ZNF154*). We evaluated the performance of individual genes and a combined panel, reporting sensitivity, specificity, positive predictive value (PPV), negative predictive value (NPV), area under the receiver operating characteristic curve (AUC and ROC), and 95% confidence intervals (CI).

**Results:**

One hundred and seventy-two subjects (86 cases and 86 controls) with a mean age of 60.24 ± 12.54 and 59.70 ± 12.75 years old were included. *TRPS1* showed the highest sensitivity at 0.97 with specificity 0.791, yielding PPV 0.82 and NPV 0.97; *HAND2* maintained strong sensitivity (0.95) but lower specificity (0.56), with PPV 0.68 and NPV 0.92; and *ZNF154* had sensitivity 0.93 with the lowest specificity (0.35), corresponding to PPV 0.59 and NPV 0.83. A combined methylation assay where all three genes are methylation-positive significantly improved specificity to 0.95 while maintaining acceptable sensitivity (0.86), resulting in the highest positive predictive value (0.95) and a robust negative predictive value (0.87). The ROC analysis showed strong diagnostic performance for all three urinary methylation markers: *TRPS1* had the highest AUC at 0.98 (95% CI: 0.96–0.99), followed by *HAND2* at 0.90 (95% CI: 0.85–0.95) and *ZNF154* at 0.82 (95% CI: 0.76–0.89).

**Conclusions:**

A urine−based three−gene methylation panel (*TRPS1*, *HAND2*, *ZNF154*) demonstrates promising accuracy for bladder cancer detection, with complementary signals improving specificity. Findings are constrained by a single–center cohort and moderate size and require external, multi−center validation in the future.

## 1 Introduction

Urothelial carcinoma is one of the most common malignancies of the urinary tract, encompassing both upper tract urothelial carcinoma (including cancers of the renal pelvis and ureter) and bladder cancer (1). Notably, bladder cancer accounts for over 90% of all urothelial carcinoma cases, with approximately 573,000 new diagnoses and 213,000 deaths reported globally each year (2). Its incidence is significantly higher in men than in women, with a male-to-female ratio of approximately 4:1 (2, 3). Bladder cancer is thus recognized as one of the most prevalent and lethal cancers worldwide, characterized by a multifocal origin, high recurrence rates, aggressive invasiveness, and frequent resistance to therapy (4).

Currently, the clinical diagnosis of bladder cancer relies primarily on cystoscopy combined with histopathological biopsy (5). Although cystoscopy with biopsy is widely regarded as the diagnostic gold standard, it is inherently invasive; patient adherence may be compromised by discomfort and pain, and the procedure is costly, imposing burdens on healthcare systems and patients alike (6, 7). Approximately 75% of patients present with non–muscle-invasive bladder cancer (NMIBC) at initial diagnosis, a subtype with high recurrence rates that necessitates intravesical therapy and regular cystoscopic surveillance (8, 9). By contrast, muscle-invasive bladder cancer (MIBC) carries a substantial risk of metastasis, and even after radical cystectomy, the five-year overall survival remains roughly 50%–60% (5). These unfavorable outcomes underscore the urgent need to implement and scale early screening and early diagnosis for bladder cancer. Early detection is essential to improve survival and cure rates; however, accurate noninvasive diagnostic modalities remain limited in current practice.

The occurrence of bladder cancer directly affects the composition of blood and urine. As a body fluid that is in direct contact with tumors of the urinary system, urine has become one of the primary focuses for noninvasive testing research (6, 10). Although urinary cytology is widely used in clinical practice, its sensitivity is relatively low, particularly for identifying low-grade tumors, and it is highly dependent on the examiner, with substantial subjectivity (11). Multiple urinary tumor biomarkers, such as NMP22 and BTA-TRAK, have been introduced to clinical practice; however, their diagnostic accuracy and clinical utility remain controversial (6). Recent studies have shown that aberrant methylation of tumor-related genes is an important molecular mechanism in the initiation and progression of urologic malignancies (12, 13). As a molecular-level diagnostic approach, DNA methylation biomarkers can reflect epigenetic alterations in genes and are amenable to detection. Existing research has found that methylated CpG sites in urine show promise for bladder cancer surveillance. For example, the 15-marker Bladder EpiCheck™ test demonstrates a specificity of 88.0% and a sensitivity of 68.2% for NMIBC (14). A company has developed UriFind, a noninvasive early screening test for bladder cancer that measures methylation levels of two genes in DNA from exfoliated urinary cells. In patients with suspected bladder cancer, the test demonstrated a sensitivity of 88.1% and a specificity of 89.7% (15). These results underscore the strong potential of urinary DNA methylation assays for bladder cancer diagnosis. However, further methodological refinements and validation in large, multicenter cohorts across Asia are still required.

The primary objective of this study is to evaluate the diagnostic performance of a novel assay that detects methylation of three urinary gene markers—*TRPS1*, *HAND2*, and *ZNF154*—for bladder cancer. Specifically, we aim to define the diagnostic value and feasibility of urinary gene methylation testing and to assess its clinical applicability. The findings are intended to provide a scientific basis for advancing noninvasive, precise screening and longitudinal surveillance of urothelial tumors.

## 2 Methods

### 2.1 Research design and study subjects

**Figure 1:**
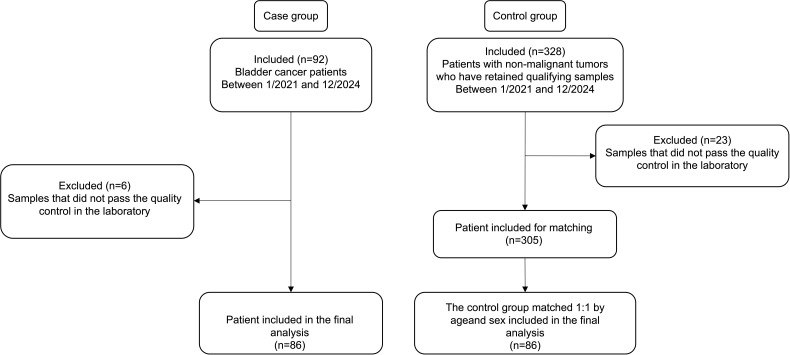
Flowchart of study participant enrollment.

This study employed a case-control design and recruited two populations at Haicang Hospital from January 2021 to December 2024. As outlined in **Figure 1**, individuals presenting to Haicang Hospital with a confirmed diagnosis of bladder cancer were included as the case group, while individuals presenting to the same hospital during the same period with symptoms such as hematuria or lower urinary tract symptoms, in whom bladder cancer and other urinary tract tumors were excluded by imaging and/or cystoscopy, were enrolled as controls. All participants met the following inclusion criteria: age ≥ 18 years; ability to provide a urine specimen; and provision of written informed consent. Exclusion criteria included menstrual urine, urine collected less than 3 hours after the previous void, and insufficient urine volume for testing. Finally, participants were enrolled and allocated according to the screening flow shown in the figure, and demographic and clinical data were recorded. The study was approved by the Medical Ethics Committee of Haicang Hospital in Xiamen (approval No. KY-2022003), and informed consent was obtained from all participants.

### 2.2 Information collection and processing

Researchers collected detailed clinical data from patients, including demographics, medical history, physical examination findings, imaging studies, painless flexible cystoscopy with biopsy pathology, and relevant information on medication and surgical treatment outcomes. Urine samples were also collected. Patients provided a random urine specimen (100–200 mL) using a 250 mL sterile cup, which was then transferred to a dedicated collection tube and stored at −80 °C until testing. All participants underwent NBI fluorescence flexible cystoscopy with biopsy for pathological examination, and the final diagnosis was confirmed by integrating imaging findings (ultrasound/MRI) and clinical treatment records.

Exfoliated cells were enriched from 100–200 mL of urine by centrifugation, and genomic DNA was extracted using the QIAamp DNA Mini Kit. Bisulfite conversion was performed with the EZ DNA Methylation-Gold™ Kit, followed by PCR amplification. Urine specimens were collected prior to cystoscopy or any surgical intervention and processed within 4 hours of collection. Samples were centrifuged at 3,000 rpm for 10 minutes at 4 °C. To minimize leukocyte DNA contamination attributable to hematuria, samples with visible gross hematuria were deferred and collected no earlier than one week after any prior cystoscopic procedure. DNA concentration was quantified prior to bisulfite conversion, and samples were stored at −80 °C with no more than three freeze-thaw cycles to ensure DNA integrity.

Methylation analysis targeted the promoter-associated CpG islands of TRPS1 (chr8q24.12), HAND2 (chr4q33), and ZNF154 (chr19q13.43) using bisulfite-converted DNA as template for quantitative methylation-specific PCR (qMSP). A sample was classified as methylation-positive for each gene if the qMSP signal exceeded the threshold established by ROC analysis of the control group. Each batch of reactions included a fully methylated positive control (M.SssI-treated DNA) and an unmethylated negative control (normal leukocyte DNA). All methylation analyses were performed by laboratory personnel blinded to the case/control status of the samples. Intra-assay reproducibility was assessed by performing duplicate qMSP reactions for all samples, with an acceptable intra-assay variation defined as ΔCt < 1, and a standard calibration curve was constructed using serially diluted *in vitro*-methylated DNA (R² ≥ 0.99 across at least four Ct points).

### 2.3 Statistical analysis

Continuous variables are described using mean ± standard deviation (SD), and categorical variables are described using frequency and percentage. Paired *t*-tests were used to compare the distribution differences of continuous variables between the case and control groups. We employed conditional logistic regression models to generate predicted probabilities for constructing the receiver operating characteristic (ROC) curves. ROC curves were generated and areas under the curve (AUCs) were estimated with 95% confidence intervals (CIs) via the nonparametric DeLong method. We calculated sensitivity, specificity, positive predictive value (PPV), and negative predictive value (NPV) with 95% CI for each gene marker. For the panel denoted “Methylation,” we used a strict AND rule whereby a sample was classified as positive only if all three genes (*TRPS1*, *HAND2*, and *ZNF154*) were methylation-positive; otherwise, it was classified as negative. All tests were two-sided with *α* = 0.05. We used R (version 4.4.1) for data cleaning and computation.

## 3 Results

As shown in **Table 1**, a total of 172 participants were enrolled, including 86 bladder cancer cases and 86 controls. The mean age was comparable between groups (cases: 60.24 ± 12.54 years; controls: 59.70 ± 12.75 years; *P* = 0.31). Since the case and control groups in this study were matched for gender, the gender distribution was the same in both groups, including 62 males (72.00%) and 24 females (28.00%). Overall, no significant differences in baseline demographics were observed between cases and controls.

**Table 1:** Baseline characteristics of the study population.

Characteristic	Case group (*N* = 86)	Control group (*N* = 86)	*P* value
Age			
Mean ± SD	60.24 ± 12.54	59.7 ± 12.75	0.308^a^
Gender			-
Male	62 (72%)	62 (72%)	
Female	24 (28%)	24 (28%)	

^a^ Paired *t*-test.

Among 86 patients with bladder cancer, tumors were slightly more often left−sided than right−sided (47.67% left *vs.* 37.21% right), with 15.12% bilateral. Lesions most commonly involved the dome and posterior wall (each 19.77%), followed by multifocal disease (18.60%), the trigone and lateral wall (each 15.12%), and the anterior wall (11.63%). Hydronephrosis was present in 31.40% of cases. The mean tumor size was 3.00 ± 1.61 cm. Histologically, pure urothelial carcinoma predominated (82.56%), while urothelial carcinoma with squamous differentiation accounted for 10.47% and with glandular differentiation for 6.98%. Lymphovascular invasion was identified in 9.30% of tumors. By T stage, 46.51% were Ta, 23.26% T1, 15.12% T2, 8.14% T3, and 6.98% T4. Lymph−node metastasis was documented in 10.47% of patients. High−grade tumors were common (70.93%), and carcinoma *in situ* was observed in 18.60%. Single lesions were more frequent than multiple lesions (61.63% *vs.* 38.37%). These results are shown in **Table 2**.

**Table 2:** Pathological characteristics of bladder cancer in the case group.

Characteristic	Case group (*N* = 86)
Tumor laterality	
Bilateral	13 (15.12%)
Right	32 (37.21%)
Left	41 (47.67%)
Tumor location	
Lateral wall	13 (15.12%)
Dome	17 (19.77%)
Multifocal	16 (18.60%)
Posterior wall	17 (19.77%)
Anterior wall	10 (11.63%)
Trigone	13 (15.12%)
Hydronephrosis	
Yes	27 (31.40%)
No	59 (68.60%)
Tumor size (cm)	
Mean ± SD	3.00 ± 1.61
Histology	
Pure urothelial carcinoma	71 (82.56%)
Urothelial carcinoma with squamous differentiation	9 (10.47%)
Urothelial carcinoma with glandular differentiation	6 (6.98%)
Lymphovascular invasion (LVI)	
Yes	8 (9.30%)
No	78 (90.70%)
T stage	
Ta	40 (46.51%)
T1	20 (23.26%)
T2	13 (15.12%)
T3	7 (8.14%)
T4	6 (6.98%)
Lymph node metastasis	
Yes	9 (10.47%)
No	77 (89.53%)
Tumor grade	
Low grade	25 (29.07%)
High grade	61 (70.93%)
Carcinoma *in situ* (CIS)	
Yes	16 (18.60%)
No	70 (81.40%)
Tumor multiplicity	
Single	53 (61.63%)
Multiple	33 (38.37%)

**Figure 2:**
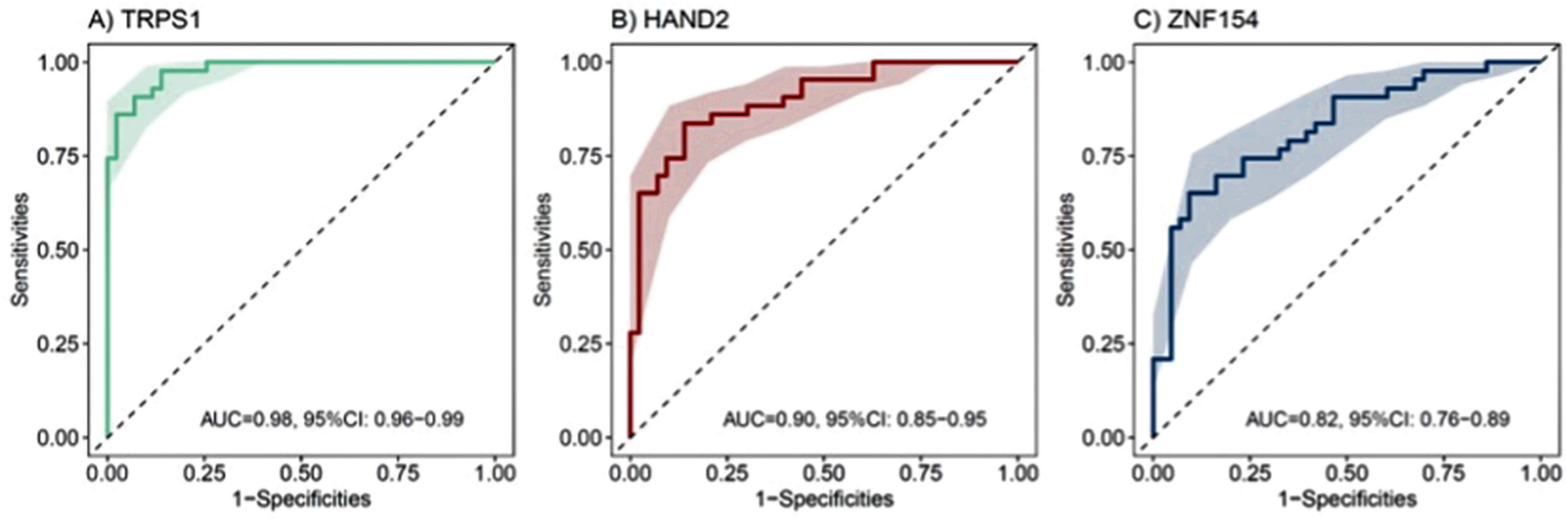
Receiver operating characteristic (ROC) curve of methylation assay for bladder cancer prediction. **(A)** ROC curve of TRPS1 with an AUC of 0.98 (95% CI: 0.96–0.99), **(B)** ROC curve of HAND2 with an AUC of 0.90 (95% CI: 0.85–0.95) and **(C)** ROC curve of ZNF154 with an AUC of 0.82 (95% CI: 0.76–0.89).

As shown in **Figure 2**, receiver operating characteristic analyses of the three urinary methylation markers demonstrated robust diagnostic performance for bladder cancer: *TRPS1* (**Figure 2A**) achieved an AUC of 0.98 (95% CI: 0.96–0.99), *HAND2* (**Figure 2B**) yielded an AUC of 0.90 (95% CI: 0.85–0.95), and *ZNF154* (**Figure 2C**) showed an AUC of 0.82 (95% CI: 0.76–0.89).

As summarized in **Table 3**, each single-gene assay showed high sensitivity but variable specificity. *TRPS1* achieved the highest sensitivity at 0.98 with specificity 0.79, yielding PPV 0.82 and NPV 0.97. *HAND2* maintained strong sensitivity (0.95) but lower specificity (0.56), with PPV 0.68 and NPV 0.92. *ZNF154* presented sensitivity 0.93 and the lowest specificity (0.35), corresponding to PPV 0.59 and NPV 0.83. Notably, the combined methylation panel improved specificity to 0.95 while retaining acceptable sensitivity (0.86), resulting in the highest PPV (0.95) and a robust NPV (0.87). Given the 1:1 matched design, PPV and NPV reflect an artificial prevalence of 50%; at a surveillance-representative prevalence of 20–30% (16), the adjusted PPV and NPV of the combined panel are estimated at 0.79 and 0.95, respectively (17). Concurrent urinary cytology demonstrated a sensitivity of 0.48 and specificity of 0.99, with PPV of 0.98 and NPV of 0.65. Sensitivity was 0.84 in high-grade tumors and 0.28 in low-grade tumors. These results are summarized in **Table 3**.

**Table 3:** Predictive performance of methylation testing for bladder cancer detection.

Methylation	Case group (*N* = 86)	Control group (*N* = 86)	Sensitivity	Specificity	PPV	NPV
TRPS1						
Negative	2 (2.3%)	68 (79%)	0.977	0.791	0.824	0.971
Positive	84 (98%)	18 (21%)				
HAND2						
Negative	4 (4.7%)	48 (56%)	0.953	0.558	0.683	0.923
Positive	82 (95%)	38 (44%)				
ZNF154						
Negative	6 (7.0%)	30 (35%)	0.930	0.349	0.588	0.833
Positive	80 (93%)	56 (65%)				
Methylation						
Negative	12 (14%)	82 (95%)	0.860	0.953	0.949	0.872
Positive	74 (86%)	4 (4.7%)				
Cytology						
Negative	45 (52%)	85 (99%)	0.477	0.988	0.976	0.654
Positive	41 (48%)	1 (1%)				

PPV, Positive Predictive Value; NPV, Negative Predictive Value.

To further evaluate the clinical utility of the panel across pathological subgroups, sensitivity was assessed within each subgroup of the 86 bladder cancer cases. The three-gene panel maintained high sensitivity across all subgroups: NMIBC (0.85) and MIBC (0.89); high-grade tumors (0.90) and low-grade tumors (0.76); CIS-present (0.94) and CIS-absent (0.84) cases. Notably, in low-grade Ta tumors — the subgroup where urinary cytology performs most poorly — the panel achieved a sensitivity of 0.75, approximately three times the concurrent cytology sensitivity of 0.25 observed in this subgroup.

To further corroborate the biological validity of the three selected markers, we performed an independent validation analysis using publicly available data from the The Cancer Genome Atlas-Bladder Urothelial Carcinoma (TCGA-BLCA) cohort (412 bladder cancer tissue samples and 21 adjacent normal tissue samples profiled on the Illumina HumanMethylation450 array). All three genes demonstrated statistically significant promoter hypermethylation in bladder cancer tissue compared with adjacent normal tissue: TRPS1 (mean beta value: 0.24 vs. 0.16, p < 0.001), HAND2 (mean beta value: 0.51 vs. 0.24, p < 0.001), and ZNF154 (mean beta value: 0.63 vs. 0.38, p < 0.001). ROC analysis demonstrated that the three-gene combined panel achieved an AUC of 0.96 (95% CI: 0.92–0.99), outperforming each individual marker (TRPS1: AUC = 0.72, 95% CI: 0.66–0.77; HAND2: AUC = 0.89, 95% CI: 0.83–0.96; ZNF154: AUC = 0.93, 95% CI: 0.91–0.96). DeLong tests confirmed that the panel AUC was statistically superior to TRPS1 (p < 0.001) and HAND2 (p = 0.002), while the difference between the panel and ZNF154 did not reach statistical significance (p = 0.093).

## 4 Discussion

In this single-center case-control study, we evaluated urinary DNA methylation of three genes (*TRPS1*, *HAND2*, and *ZNF154*) for bladder cancer detection. We found each single-gene assay demonstrated high sensitivity with varying specificity, with *TRPS1* performing best among individual markers (sensitivity 0.98; specificity 0.79). Applying a strict AND rule to form a composite “Methylation” panel (positive only when all three genes were methylation−positive) markedly increased specificity to 0.95 while maintaining acceptable sensitivity at 0.86, yielding the highest PPV (0.95) and a robust NPV (0.87). These findings indicate that the urine-based methylation panel has strong diagnostic potential and could complement cystoscopy for noninvasive triage and surveillance.

Direct comparison with concurrent urinary cytology further contextualizes the clinical utility of our panel. Cytology achieved near-perfect specificity (0.99) but limited overall sensitivity (0.48), with particularly poor performance in low-grade tumors (0.28). Our three-gene methylation panel demonstrated substantially higher sensitivity (0.86) and NPV (0.87) while maintaining comparable specificity (0.95). The complementary performance profiles suggest potential synergy within a tiered diagnostic algorithm, where methylation testing serves as an initial sensitive screen and cytology as confirmatory evaluation — an approach particularly relevant for low-grade NMIBC surveillance where cytology has historically been inadequate. This synergistic approach is consistent with emerging evidence demonstrating that the simultaneous use of methylation-based assays and urinary cytology can improve both the sensitivity and specificity of urothelial lesion follow-up beyond what either modality achieves alone, supporting their integration within a complementary diagnostic framework rather than as mutually exclusive alternatives (18–20).

Our findings are consistent with, and in several aspects comparable to, prior urine−based methylation assays for bladder cancer. Compared with urine cytology, which is highly specific but has modest sensitivity, especially for low−grade disease, methylation testing generally offers higher sensitivity with acceptable specificity (21, 22). Commercial panels such as Bladder EpiCheck (15−marker DNA methylation signature) have reported overall sensitivities of 68.20% at specificities of 88.00%, as reported in multicenter real-world and prospective evaluations (14), while the UroMark/UrMark and other targeted methylation panels in case-control settings achieve AUCs around 0.97 (23). In our study, the best single marker, *TRPS1*, delivered higher single−gene performance than many previously reported individual loci, and the strict “Methylation” panel further improved specificity, aligning with the observation that multi−locus methylation signatures reduce false positives without a major loss of sensitivity (24).

The broader molecular landscape of bladder cancer further contextualizes the biological rationale for epigenetic-based detection. Bladder cancer exhibits significant molecular and histologic heterogeneity, with the WHO 2022 classification increasingly incorporating molecular signatures — including DNA methylation patterns — alongside morphological criteria to better characterize disease subtypes and guide therapeutic decisions (25). At the genomic level, aberrant DNA methylation represents one of the most prevalent epigenetic alterations in bladder cancer, influencing tumor suppressor gene silencing, tumor progression, recurrence, and immune evasion. DNA methylation profiles have been shown to identify prognostically distinct molecular subgroups (26), and integrative multi-omics analyses have further demonstrated the utility of methylation and hydroxymethylation signatures in characterizing recurrent disease and predicting immunotherapy response, including PD-L1 expression (27–29). Collectively, these findings underscore that DNA methylation is not merely a diagnostic signal but a functionally relevant molecular feature of bladder cancer, supporting the translational rationale for urine-based epigenetic assays as both diagnostic and potentially prognostic tools.

The biological plausibility of the three selected genes supports their utility in urine assays. *TRPS1* encodes a GATA type transcription factor implicated in epithelial differentiation; aberrant methylation and dysregulated *TRPS1* expression have been linked to urothelial carcinogenesis and aggressiveness, making it a rational, tumor derived signal detectable in shed urothelial DNA (30, 31). *HAND2* is a well characterized tumor suppressor frequently silenced by promoter hypermethylation across epithelial malignancies and has been validated as a sensitive methylation marker in gynecologic and gastrointestinal cancers, suggesting cross tumor applicability to urothelial neoplasia (32, 33). *ZNF154* is a pan cancer methylation hotspot originally identified by genome wide screens (34). Hypermethylation of *ZNF154* robustly discriminates tumor from normal across multiple tissues and has been reported in urothelial carcinoma, making it a useful component for specificity in multi marker panels (24, 35). Together, these genes complement each other. *TRPS1* shows strong stand−alone power to tell tumor from normal in our data. *HAND2* and *ZNF154* add independent methylation signals that reduce false positives and improve specificity. This explains why a strict panel rule yields better overall performance.

This study leverages a biologically grounded, complementary three−gene methylation panel in urine to improve specificity. The assays were conducted with rigorous quality control and clinically relevant samples, enhancing technical robustness and translational relevance. However, the study also has limitation. The single-center cohort and moderate sample size remain important limitations of the present study. The 1:1 matched case-control design yields an artificial disease prevalence of 50%, rendering PPV and NPV non-generalizable to real clinical settings; spectrum bias may additionally inflate performance estimates relative to unselected surveillance populations. To partially address the lack of external validation, we performed an independent analysis of the TCGA-BLCA cohort, which confirmed consistent promoter hypermethylation of all three genes in bladder cancer tissue across an independent Western population. Nevertheless, this tissue-based corroboration cannot substitute for urine-based external validation, as absolute methylation levels and assay performance may differ between sample types. Future studies will prioritize prospective, multi-center validation using urine samples with pre-specified positivity thresholds, head-to-head comparisons against established urine biomarkers and cytology, and evaluation of assay performance across diverse demographic and clinical settings to support clinical implementation. Moreover, the high AUC observed for individual markers, notably TRPS1 (0.98), should be interpreted with caution, as enriched case-control designs are known to inflate single-marker performance estimates relative to real-world clinical settings.

## 5 Conclusion

A urine-based three-gene methylation panel combining *TRPS1*, *HAND2*, and *ZNF154* shows promising accuracy for bladder cancer detection, with complementary signals improving specificity under rigorous quality control and clinically relevant sampling. The panel is primarily intended for non-invasive surveillance of patients with previously diagnosed NMIBC, a population well represented in our cohort in which Ta and T1 tumors comprised approximately 70% of cases. Findings are constrained by a single-center cohort and moderate size, and require external, multi-center validation in the future.

## Data Availability

The raw data supporting the conclusions of this article will be made available by the authors, without undue reservation.
